# Responsible Management Education in Time of Crisis: A Conceptual Framework for Public Business Schools in Egypt and Similar Middle Eastern Context

**DOI:** 10.1007/s11115-021-00532-6

**Published:** 2021-07-21

**Authors:** Mohamed Mousa, Hiba Massoud, Rami Ayoubi

**Affiliations:** 1 WSB University, Dabrowa Gornicza, Poland; 2 Cardiff Metropolitan University, Cardiff, UK; 3 Coventry University, Coventry, UK

**Keywords:** Responsible management education, Institutional theory, Stakeholder theory, Covid-19, Egypt, Middle East

## Abstract

Recent studies show that the adoption of RME scenarios is still a matter of concern for non-western countries ((Mousa et al., Journal of Management Development 38:681–696, 2019), 2021a, 2021b). In this paper, we theoretically propose the potential direction of RME scenarios that business schools in Egypt and other similar cultural context to implement through articulating the main antecedents of RME before and after Covid-19. we used the method of multilevel research by combining different theoretical approaches. As an outcome of our analysis, we developed five propositions which form the main antecedents of RME in Egypt and similar regional Middle East business schools before and after Covid-19.

## Introduction

Since December 2019, nothing has been more important in the world’s socio-political and economic agenda than the coronavirus pandemic (Covid-19). This virus has led to the death of more than 2.5 million people after by 1 March 2021 (Worldometer, 2020). It has been perceived by Khan (2020) as the most dangerous biological threat to humans in the history of the world. Carruthers (2020) highlights that the sudden emergence and later identification of this virus has assisted in its rapid spread worldwide, requiring an unusual and unexpected increase in the demand for health-care services and related industries and/or supplies. Xu et al. (2020) indicate that Corona phobia and the fear of getting infected by this virus as a serious threat that psychologists have to address. Ren et al. (2020) consider the consequences of negative prejudice against coronavirus patients as one of the unexpected outcomes of this virus. Economically, this pandemic has not only stopped work activities in both public and private organizations, such as universities, shopping malls, restaurants and factories, but also forced countries to suspend its normal and traditional life cycles or activities for long period of time. Khan (2020) considers the cuts to national sources of income, particularly those that depend on oil and tourism, as a serious threat to world peace.

From another perspective, over the past two decades, business schools have not fully exercised their pre-assumed role as a change agent for profit and not-for-profit organizations. In this context, Anninos and Chytiris (2011) elaborate that the majority of business schools equip their students only with economic and management theories that do not entail any social obligations that all organizations have. Topics such as climate change, carbon footprint, gender equality, unemployment, human rights and corruption are not listed as priorities in the research centres and curricula of business schools (Podolny, 2009; Petriglieri, 2012; Doherty et al., 2015). Consequently, Alvesson (2013) have called for business schools to revisit the content of their courses, fieldworks and exercises. Doppelt (2009) asserts that the main threat facing today’s business schools is deciding how and to what extent they are able to meet the social obligations of their stakeholders and develop environmentally friendly initiatives.

Consequently, and as a response to the aforementioned calls, accusations, and perceived extensive pressure, some business schools – particularly in the West – have started to include sustainability-related behaviour in their curricula. Others have developed some tailor-made courses on social and ethical themes, where academics have devoted considerable space to empirically incorporating CSR, corporate citizenship, societal citizenship, cultural equality, organizational inclusion among others into their research agenda (Steketee, 2009; Wu et al., 2010; Pless et al., 2011; Brower, 2011). Furthermore, universities have fostered agreements with some national and international NGOs to effectively address environmental threats and social interests. For instance, the University of Exeter in UK has initiated a “one planet MBA” programme in collaboration with a famous international NGO organization called (WWF International) primarily aims to deliver a new generation of responsible leaders, who can create, develop and disseminate virtues, wisdom and society-related knowledge into their surrounding communities. (WWF International, 2011). While the RME scenarios are quickly advancing the efforts of curricula development in the West, it is still a matter of concern for other countries in other regions including African countries and some countries in the Middle East, where previous findings show that academics in that regions still not very ready for curricula development of RME (Mousa et al., 2019), and that Covid-19 has not had any effect yet on the adoption of sustainable business education in the higher education (Mousa, 2020a), however some very recent findings show that the spread of Covid-19 has positively changed the situation and that the current socio-cultural challenges start to shape the minds of business students, academics and trainers (Mousa, 2020b).

Given the recent findings above (Mousa et al., 2019; Mousa 2021a, b), and using a multilevel view, in this paper, we elaborate and theoretically propose and predict the potential direction of RME that business schools in that region are to implement through identifying and articulating the main antecedents of RME before and after Covid-19, and considering both the information business schools should develop concerning the socio-economic and environmental impacts of Covid-19 and the change in working conditions.

## RME and the Response of Business Schools

Erskine and Johnson (2012) confirm that sustainability was the main driver behind the emergence of the 2004 principles of RME. Economic growth is perceived to be sustainable when “it meets the needs of the present generation without compromising the ability of future generations to meet their own needs” (Brundtland commission, 1987, p. 8). This indicates that sustainability not only concerns the natural environment but also human prosperity and economic development (Erskine & Johnson, 2012). Holliday (2010) describes sustainability as a novel growing trend in RME. However, Bridges and Wilhelm (2008) assert that the top 30 business schools devote 25 per cent of their curricula, research and coursework to sustainability-related aspects. Dean and Beggs (2006) highlight that many academics continuously reveal their urgent need for well-designed training in order to effectively teach social responsibility and ethics-related ideas. Accordingly, in 2004 and in an attempt to embed sustainability, ethics and social-related challenges (e.g. safeguarding human rights, fighting corruption, promoting gender equality) within management education, the United Nations (UN) launched its global voluntary initiative known as the UN Principles for Responsible Management Education (PRME). This initiative is the result of years of collaboration with business schools, accredited bodies and associations, such as the AACSB, European Foundation for Management Development (EFMD), the Academy of Business in Society and the Aspen Institute. PRME has been perceived by author such as Sterling (2012) as an opportunity for business schools to rethink the content of their courses, teaching pedagogy, research scholarships and even their engagement with stakeholders. The following table presents the principles of REM developed by the UN. Table 1

**Table 1:** UN Principles of Responsible Management Education (source:PRME, 2012)

Principle	Content
Principle 1: Purpose	We will develop the capabilities of students to be future generators of sustainable value for business and society at large and to work for an inclusive and sustainable global economy
Principle 2: Value	We will incorporate into our academic activities and curricula the values of global social responsibility as portrayed in international initiatives such as the UNGC
Principle 3: Method	We will create educational frameworks, materials, processes and environments that enable effective learning experiences for responsible leadership
Principle 4: Research	We will engage in conceptual and empirical research that advances our understanding about the role, dynamics and impact of corporations in the creation of sustainable social, environmental and economic value
Principle 5: Partnership	We will interact with managers of business corporations to extend our knowledge of their challenges in meeting social and environmental responsibilities and to explore jointly effective approaches to meeting these challenges
Principle 6: Dialogue	We will facilitate and support dialogue and debate among educators, business, government, consumers, media, civil society organizations and other interested groups and stakeholders on critical issues related to global social responsibility and sustainability

Mintzberg (2005) elaborates that the functional specialisation of MBA programmes which only export financial information is no longer sufficient to form student awareness. Moreover, focusing only on maximizing profits hinders any attempt to deliver student communication skills, leadership ability, emotional maturity and global orientation (Slater and Dixon-Fowler, 2010; Fougere et al., 2014). This has caused authors such as Ghoshal (2005) and Giacalone and Thompson (2006) to question the expected value, benefit and outcomes from only paying attention to agency and transaction costs.

## RME in the Public Business Schools in Egypt and Similar Middle East Context

According to Brookes et al. (2014), there is a shortage of empirical studies that address the implementation of RME in different business schools. However, the situation is even worse in business schools in the Middle East, and the authors concur that they also discovered a dearth of academic publications on this issue in the region. Alnodel et al. (2018) indicate that economic stability is the main determinant for Middle Eastern business schools not engaging with their surrounding stakeholders. Accordingly, neglecting RME in these schools comes as a result of the economic instability in those countries. In a Jordanian study, Abu-Alruz et al. (2018) found that students in Jordan maintain a very positive attitude towards the three axes of sustainable development – economic viability, society and education. In 2014, a regional discussion at the ESCA School of Management in Casablanca, Morocco ended with the following four challenges and considered them as the main barriers to business schools in the Middle East implementing RME: 1. Curriculum development and content sharing, 2. Research including anti-corruption; 3. Outreach on the sustainability literacy test; and 4. Women on boards in the Arab Region (Fourth PRME MENA Regional Forum, 2014) (https://www.unprme.org/how-to-engage/display-working-group.php?wgid=3168).

In the Egyptian academic context, which according to the 2017 Global Competitiveness Report (www.weforum.org/) was ranked as having the lowest quality management education, Mousa et al. (2019a) and Mousa et al. (2020), have elaborated that unlike many Western business schools, tackling global responsibility and environment-related themes such as climate change, human rights, poverty, unemployment and carbon footprints are identified absent from the agenda in Egyptian and Middle-Eastern public business schools. Moreover, if authors (e.g. Rabasso & Rabasso, 2011; Waddock et al., 2010) highlight that the inclusion of sustainability in teaching and research scholarships at business schools has become the cornerstone in all accrediting bodies, such as Association to Advance Collegiate Schools of Business (AACSB) and European Quality Improvement System (EQUIS) evaluating business schools, the matter is seen different in the Middle East. Mousa et al. (2019) indicate that public business schools in the Middle East are only accredited by the local governmental ministries of education in their countries. Accordingly, these schools can function without paying attention to or even being aware of some regulatory bodies, such as the AACSB. Such ongoing guaranteed local accreditation indicates why those business schools choose to limit their attention to lectures and course work on finance and business-related research problems in their institutes and research centres (Mousa, 2019b), which is not in line with Ackoff (2004) and Giacalone (2004), who assert the importance of social knowledge besides financial literacy in securing a future for the school and employment for their graduates. However, it is worth highlighting that all public business schools in the Middle East are funded by the governments of those countries, and the question remains on how is it possible to direct the efforts towards sustainability in business education in these schools? Understandably, Corley and Gioia (2000) and Slaughter and Rhoades (2004) link any attempt to activate RME with the sufficiency of financial capabilities and/or resources. Otherwise, any discourse about sustainable business education will look like planting in a running river.

Therefore, drawing on recent work on the socio-economic implications and environmental impact of Covid-19 (Khan, 2020; Helm, 2020; Carruthers, 2020), we seek to theorise on the direction Middle Eastern business schools can take in implementing RME. In other words, we seek to provide an answer to the following question: *What are the main antecedents stimulating the implementation of RME in Egypt and similar regional Middle-Eastern business schools before and after the emergence of Covid-19?*

## Methods of our Analysis

In this paper, we used the method of ‘multilevel research’, as our analysis goes beyond statistical techniques (Hitt et al., 2007), and consider other important elements. Multilevel research includes the development of multilevel theory by combining different theoretical approaches at different levels and establishing relationships between constructs at different levels (Molina-Azorín et al., 2019).

To develop our multilevel theory, we first turn to recent publications on managing sustainability during crisis and the emergence of infectious diseases, maintaining human resources during pandemics, and some academic works on learning for sustainability, learning from poverty and exposing values in management education (Erskine & Johnson, 2012; Fougere et al., 2014; Neal, 2017; Carruthers, 2020; Aburumman et al., 2020). Second, we integrate institutional theory (Zucker, 1987) and stakeholder theory (Ullmann, 1985a, b) into the discussion on what has motivated the initiation of RME before and since the Pandemic. Institutional theory helped the authors in entailing a discussion (before the Covid-19 pandemic) about what is appropriate and widely accepted in terms of business schools pursuing RME, while stakeholder theory urges us to account for what direction business schools should currently take in order to revitalise and modernise RME to meet the new demands and expectations of stakeholders post Covid-19.

## Conceptual Foundation

### Institutional Theory and RME

Boxenbaum and Jonsson (2008) consider such reorientation of business schools towards implementing and sustaining RME relies completely on organizational institutionalism from institutional theory. According to this theory, organizations are affected by the institutional context they operate in (Zucker, 1987). This context consists of “common understandings of what is appropriate, and fundamentally meaningful behavior” (Zucker, 1987, p. 105). According to Scott (1995), institutionalised practices should come in line withal generally accepted behaviour not only in the surrounding context but also in the global one if possible. DiMaggio and Powell (1983) elaborate that organizations adopt widely accepted institutional practices because of the following three motives: coercive isomorphism (fear of sanctions and punishments), mimetic isomorphism (imitating successful competitors/market leaders) and normative isomorphism (simply because such institutional practices are the right and sometimes sole choice to adopt). Accrediting bodies, government regulators, the UN and other providers of rankings urge business schools to adopt and maintain RME. According to Scott et al. (2000, p. 237), neo-institutional theory highlights that organizational legitimacy requires more than “material resources and technical information”.

Accordingly, efforts by business schools to adopt and maintain RME reflect their intention to regain organizational legitimacy (Scott, 1995). The pressure different stakeholders exert on business schools to update, modernise and reconsider their pedagogical mechanisms, course content and research has encouraged business schools in the direction of RME (Kurland et al., 2010). Moreover, Doherty et al. (2015) have pointed out that the following four pressures stand behind the tendency for British business schools to adopt RME: societal pressure and its resulting calls for business schools to develop a socio-moral orientation in both undergraduate and postgraduate students, pressure from businesses and calls for business schools to cultivate their curricula and to include sustainability-related aspects as core elements, pressure from accrediting and regulatory bodies (e.g. AACSB, EQUIS) to ensure the inclusion of RME in the teaching pedagogies and research scholarships at business schools, and finally institutional pressure, particularly considering that 57 per cent of students in the UK would like sustainability to be a main part of their course while 42 per cent consider that teaching sustainability-related aspects will assist them in finding employment.

#### Academic Motives

Accrediting bodies such as AACSB and EQUIS have also played a role in efforts among business schools towards sustainability. For instance, AACSB International (2004, p. 9) has requested business schools to “renew and revitalize their commitment to the centrality of ethical responsibility at both individual and corporate levels,” while EQUIS tends to design tools to assess how and to what extent values and skills are developed by business schools. Based on the latter, we make the following proposition.

*Proposition 1: Prior to Covid-19, the pressure exercised by accrediting bodies was perceived at both the managerial and individual academician level as an antecedent for RME in Egyptian and similar Middle Eastern business schools.*

#### Pressure from Businesses

According to Kashyap et al. (2006), the shift in research and education in business schools towards socially responsible practices is in response to ongoing calls from different firms to prepare graduates who possess socio-ethical and responsible mindsets. Those firms also have to respond to growing pressure from stakeholders urging firms not only to undertake more sustainable behaviour but also to reveal their social and environmental investments periodically if not even monthly (Bennis & O’Toole, 2005; Moosmayer, 2015). However, Grayson (2010) elaborates that efforts by PRME and the UN to cultivate management and business education has come late even after the inclusion of sustainability practices and components into the mission and practices of big corporations such as Nestle, Wal-Mart, Nike and Kraft, among others. Gioia (2002) and Ghoshal (2005) have touched observed at least a decade (1990–2000) of ongoing invitations from different stakeholders to reduce opportunistic behaviour and focusing only on maximizing shareholder profits and seeing the world in dollars and euros. Our second proposition is as follows:

*Proposition 2: Prior to Covid-19, the pressure exercised by businesses was perceived at both the managerial and individual academician level as an antecedent for RME in Egyptian and similar Middle Eastern business schools.*

#### The Effect of Poverty

Thirteen per cent (13%) of the world population live on less than 1.25 US dollars a day, as indicated by the World Bank (2016). This occurs despite the Millennium Development Goal (MDG) launched by the UN in 1990 to alleviate poverty (Neal, 2017). Moreover, in September 2015, the UN announced and decreed the second wave of Sustainable Development Goals (SDG), which included the following objective: “By 2030, (to) eradicate extreme poverty for all people everywhere, currently measured as people living on less than $1.25 a day… and reduce at least by half the proportion of men, women and children of all ages living in poverty in all its dimensions according to national definitions”. However, in countries such as Burundi and Madagascar, the poverty rate exceeds 77%, while in Bangladesh and some South Asian countries, the poverty level revolves around 43% (World Bank, 2016). The question that should be raised here is: Can business schools in these poor countries play a role in decreasing such poverty rates? And according to Neal (2017), the answer is yes, as business schools are the main producers of the managers, employees and other classes of decision-makers of tomorrow, who can shape the future of the poor in their countries. Moreover, poverty itself is an outcome of corruption, unethical behaviour, the unfair distribution of wealth, besides some ineffective managerial practices such as organizational nepotism, gender inequality and opaque levels of financial disclosure that negatively impact people lives. Therefore, we propose:

*Proposition 3: Prior to Covid-19, poverty was perceived by both managers and individual academicians as an antecedent for RME in Egyptian and similar Middle Eastern business schools.*

### Stakeholder Theory, Covid-19 and RME

Authors such as Davis (2006) and Chan (2014) highlight that nothing can hinder economic development and social prosperity more than the spread of bacteria and viruses, which traditionally originate or find a welcome home in unsanitary high-density localities developed and utilized by poor, uneducated people. This may explain the spread of Ebola epidemics in West Africa (Chan, 2014). However, the situation has become even worse after the emergence and spread of the Coronavirus epidemic in both rich developed and poor developing countries. As a rapid socially responsible response to this crisis, some pharmaceutical companies have announced that they will offer the components for any planned vaccines they develop to their competitors without consideration for intellectual property rights and/or financial return. Noticeably, education is one of the main sectors that has been negatively affected by Covid-19. Students feel disturbed and both universities and schools have had to close their doors. Moreover, the spread of Covid-19 has raised questions regarding the future of some educational programmes, especially business-related majors where internships and training is prioritised. Unfortunately, this question has no answer within the outbreak for academia, governments and civil society organizations.

Garriga and Mele (2004) categorise CSR theories into four groups: first, instrumental theories, in which the social behaviour of all organisations and all practices reflect nothing except a means to maximize economic returns for those organisations. Second, political theories, in which organizations employ their social responsible activities to exercise some pressure over governments and display the level and scope of power they have. Third, integrative theories, in which organizations feel they must understand societal obligations required of them and accordingly exert efforts to fulfil them. Fourth, ethical theories, in which organizations feel that social responsibility is a moral obligation they should guarantee for their societies and/or communities. According to Forray and Leigh (2012), current and previous literature and/or theories on CSR has contributed to the development and then implementation of RME.

In explaining stakeholder theory, Ullmann (1985a, b) highlights that organizations affect not only the economic and/or business market they target but also a broad increase in stakeholder groups, such as social actors, government institutions, suppliers and customers, among others. Moreover, Mitchell et al. (1997) indicate that each stakeholder group can exert powerful pressure over organizations regardless of formal structures, types and objectives. Accordingly, Lehman (1995) and Adler and Milne (1997) point out that organizations have to understand and monitor their different stakeholder groups in order to effectively meet their demands. Moreover, organizations should secure a disclosed and publicly revealed social role besides their traditional activities in maximizing shareholder wealth or guaranteeing services to clients (Gray et al., 1995). The CSR concept transitioned significantly to alternative themes such as stakeholder theory, business ethics theory, corporate social performance and corporate citizenship (Garriga,^ 2009^, P. 292).

#### The need for More Information and/or Scenarios

Helm (2020) has pondered the impact of the economic shock from the current crisis. In other words, what is the expected socio-economic and environmental impact of Covid-19 in both the short and long term? Furthermore, what is the expected impact of this virus on globalisation? The authors of the present paper also add the following questions: Are business schools ready to address the socio-economic and environmental consequences of Covid-19? And if so, are they ready to address its impact in their local surroundings or in the global arena or both? Moreover, are business schools in the developing nations including Egypt and Similar Middle Eastern countries able to tackle this? Are they thinking about re-formulating their own work agenda? Will they expect a new invitation from the UN to act and respond? Will the governments of the Middle East take RME seriously and assist their public business schools? Are academics in the public business schools of the Middle East ready to re-orient their curricula, teaching and research paradigms? Lee et al. (2016) and Rudd et al. (2018) point out that during crises and natural disasters, nothing remains important than information management and psychological treatment. Hence, we propose:

*Proposition 4: Post Covid-19, yielding and managing valuable information regarding the expected social, economic and political impact of Covid-19 is perceived, at the level of both managers and individual academicians, as an antecedent for implementing RME in Egyptian and similar Middle Eastern business schools.*

#### Changing Nature of Work Activities

Since its emergence in the Wuhan province of China in late 2019, and its worldwide spread, Covid-19 has locked down the economic activities of hospitality industries, retail stores and more. Accordingly, people have had to stay indoors (Helm, 2020). Some leading world corporations, such as Google, YouTube and Microsoft, have convinced their employees to work from home (Aburumman et al., 2020). Moreover, some organizations have started to prepare a detailed urgent career planning-related strategy to fill the gap they face after losing some valuable staff to Covid-19 deaths and/or the changing working conditions. Under this scenario, the authors believe that RME can assist business schools in preparing their graduates for the changes in working conditions. Hence, we propose:

*Proposition 5: Changing work conditions post Covid-19 is perceived at the level of both managers and individual academicians as an antecedent for implementing RME in Egyptian and similar Middle Eastern business schools.*

## Discussion

**Fig. 1:**
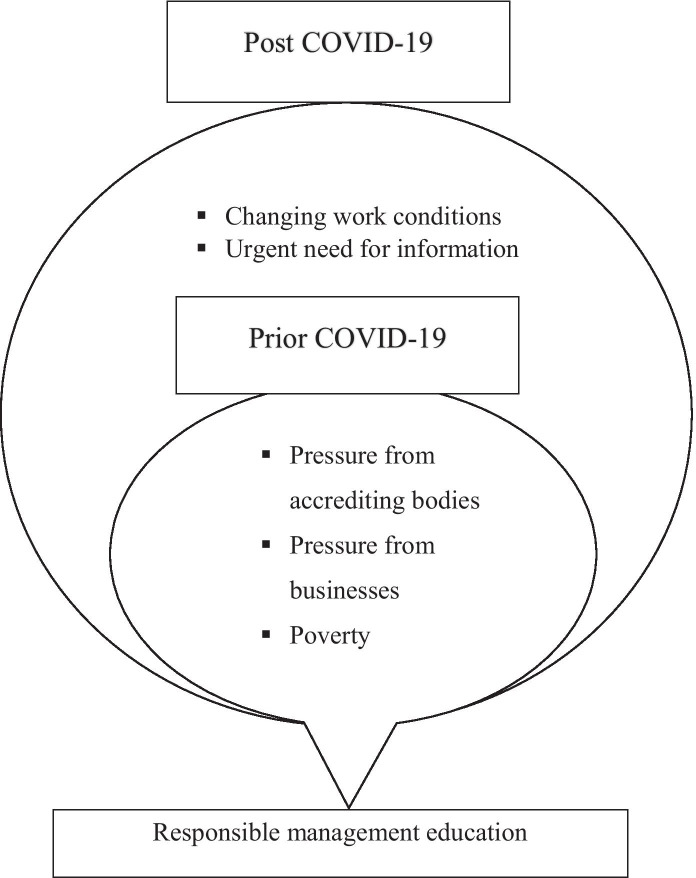
The Main Antecedents of RME in Egypt and Similar Regional Middle East Business Schools Before and after Covid-19 (composed by the authors)

The authors’ propositions here are consolidated and summarised in Fig. 1, which elaborates the main antecedents of RME prior to Covid-19 and how the identification of Covid-19 prioritises the needed information regarding the socio-economic impact of Covid-19 and changing work conditions as two new antecedents for RME.

In our paper we explored how will RME be affected by the identification of Covid-19, and what are the main antecedents of RME particularly after the emergence of Covid-19. Drawing on recent literature on management education (Fougere et al., 2014; Moosmayer et al., 2019; Parkes et al., 2017; Décamps et al., 2017), poverty (Neal, 2017), learning opportunities for sustainability (Erskine & Johnson, 2012) and on sustainability in the areas of infectious diseases (Aburumman et al., 2020; Carruthers, 2020; Khan, 2020), we advance a multilevel theoretical analysis that identifies the main antecedents for RME before and after Covid-19. Through focusing on institutional theory (Zucker, 1987), we explore how the widely accepted institutional practices derived from coercive isomorphism (fears of sanctions) and/or mimetic isomorphism (imitating successful competitors) and/or normative isomorphism (adopting the right practices) promote the implementation of RME. Our analysis proceeded via stakeholder theory, which suggests that business schools should not only prepare their students to lead socially adult lives but also to exert a continuous effort in meeting the social obligations and environmental priorities of their stakeholders. In doing so, this study secures a theoretical and practical contribution to academic literature concerned with management education.

## Theoretical Implications

Our first theoretical contribution lies in a multilevel analysis addressing the antecedents that affect RME before and after Covid-19. Considering Boxenbaum and Jonsson (2008) and the AACSB International (2004) to highlight that the continuous calls and even pressure from accreditation bodies such as EQUIS and the AACSB were supposed to drive the efforts of business schools in Egypt and similar countries in the Middle East towards sustainability and a reliance on the principles of RME. We join authors such as Bennis and O’Toole (2005), Ghoshal (2005), Kashyap et al. (2006), Grayson (2010) and Moosmayer (2015) in emphasizing the active role different businesses play in activating the adoption of sustainable business education by Western business schools. However, whether business schools in Egypt and similar Middle Eastern context have received calls from employers regarding undertaking responsible education and whether they responded or not was not one of the objectives this paper addressed. We extend the analysis of the main motives and/or factors affecting the implementation of RME by confirming that poverty, which is a barrier to economic prosperity in many developing nations (World Bank, 2016), was also supposed to stimulate business schools as the main producers of tomorrow’s policy makers and decision-makers (Neal, 2017) to utilize RME as a paradigm in fighting against nepotism, corruption and other poor managerial practices exercised in their business settings.

Our second theoretical contribution lies in expanding the discourse on RME and its presumed roles post Covid-19. Since business schools should act as change agents for commercial and non-governmental organizations, we highlight the need for business schools in Egypt and similar Middle Eastern countries to create, develop and maintain research-driven programmes that may facilitate coping with the social, economic and political impacts of the Coronavirus pandemic. We also come in line with Helm (2020) in questioning the environmental consequences of Covid-19 in both the short and long run. We shed light on the changing working conditions in some leading corporations, such as Google, YouTube and Microsoft, and the growing trend of inviting employees to work from home (Aburumman et al., 2020). This should encourage business schools to revisit what they teach their students as crucial elements for career planning, career success and entrepreneurship.

Our third contribution lies in extending the theoretical scope of stakeholder theory by Ullmann (1985a, b) by showing that in the context of a crisis, stakeholders’ needs might change and hence the organization should regularly redefine its pool of social obligations. In our case, given the crisis the labour market is currently passing through, business schools should employ RME to redefine and re-explore and then balance their students’ needs according to the changed requirements in society (Garriga, 2009; Mintzberg, 2005; Rabasso & Rabasso, 2011; Mousa et al., 2020). Accordingly, focus should be given to some educational programmes while others should be suspended or pared back.

## Future Research

This article opens up several research opportunities. First, this study calls on other interested researchers to empirically investigate the propositions posed here by the authors of this paper in different business schools, and accordingly determine the main antecedents for RME across cultures. In this regard, others may choose to empirically address our propositions in the context of a group of business schools in a single country through a qualitatively holistic case study (Gelman & Hill, 2006), or they may proceed with a multi-case design that compares the main antecedents of RME in business schools across countries (Eisenhardt, 1989).

Some researchers may find themselves interested in determining what outcomes Covid-19 may have in the context of business schools. In this case, researchers could either adopt a theoretical multilevel analysis similar to the present paper, or proceed with an empirical exploration of such outcomes through a qualitative study in a number of business schools. In addition, the effect of Covid-19 on the labour market or in other words the association between Covid-19 and changing work conditions may also be perceived as a preferable research opportunity for human resources management scholars who can emphasize the effect of this virus on personnel selection, career planning, talent management, performance appraisal and promotion opportunities.

## Managerial Implications

Given the rapid worldwide spread of Covid-19 and the pessimistic expectations regarding some viruses in the near future, we suggest that business schools in Egypt and similar Middle Eastern countries establish units for sustaining RME. This unit will maintain a systemic set of practices to ensure academics understand what is appropriate and what is expected of them (Mousa et al., 2019). This will secure widely accepted ethical academic practices and contribute to the process of rebuilding the legitimacy of business schools (Scott et al., 2000). The authors of the present paper consider that the main objective of such a unit lies primarily in producing academic research that include information and suggestions for dealing with the socio-economic and environmental impact of Covid-19 (Helm, 2020; Lee et al., 2016; Rudd et al., 2018). Furthermore, this unit should manage a zone for collaboration between business schools with different stakeholders, who may assist in mitigating the huge negative outcomes of Covid-19 on the future of those schools, and on a global context, the focus on Middle-Eastern business schools, which remains an little-known context, facilitates the mission of Western business schools seeking either to establish research cooperative activities in the Middle East or develop partnerships with educational bodies and/or civil society associations.
